# Carbon nanoparticle-entrapped macroporous Mn_3_O_4_ microsphere anodes with improved cycling stability for Li-ion batteries

**DOI:** 10.1038/s41598-022-16383-0

**Published:** 2022-07-14

**Authors:** Takahiro Kozawa, Fumiya Kitabayashi, Kayo Fukuyama, Makio Naito

**Affiliations:** Joining and Welding Research Institute, Osaka University, 11-1 Mihogaoka, Ibaraki, Osaka 567-0047 Japan

**Keywords:** Batteries, Design, synthesis and processing

## Abstract

Manganese oxide (Mn_3_O_4_) has garnered substantial attention as a low-cost, environment-friendly anode material. It undergoes a conversion reaction involving the formation of Li_2_O and metallic Mn to provide high-energy Li-ion batteries. However, its low electrical conductivity and significant volume change reduce its capacity during the initial lithiation/delithiation, hindering its practical application. To improve the cycle performance, we propose a new composite structure wherein we entrap carbon nanoparticles in macroporous Mn_3_O_4_ microspheres with a unique maze-like porous interior. We fabricate the Mn_3_O_4_/C composites using a scalable two-step process involving the thermal decomposition of MnCO_3_ in water vapor and mixing in a carbon-dispersed solution. The fabricated Mn_3_O_4_/C composites with varying carbon contents exhibit a high maximum discharge capacity retention of 86% after 50 cycles, compared to the 18% given by bare Mn_3_O_4_. The entrapped carbon nanoparticles improve the cycle performance both electrochemically and physically. The microstructure of the composite particles and the fabrication process developed in this study will help improve the performance of other conversion-type anode materials that suffer from cycle degradation, including inexpensive transition metal oxides and sulfides.

## Introduction

Mn_3_O_4_ is widely used as a sensor, catalyst, capacitor, and magnetic material because of its non-toxicity, low environmental load, stable physicochemical properties, and abundant Mn resources^1–7^. It has also gained considerable attention as an anode material for Li-ion batteries^8–10^, as it permits the following electrochemical conversion reaction: Mn_3_O_4_ + 8Li^+^  + 8e^−^  ↔ 4Li_2_O + 3Mn. The lithiation process leads to the formation of Li_2_O and metallic Mn, whereas delithiation gives Mn_3_O_4_ by reverse oxidation. The Mn_3_O_4_ anode yields a theoretical capacity of 937 mAh/g based on this conversion reaction, which is higher than that of a traditional intercalation-type anode, such as graphite (372 mAh/g). Furthermore, Mn-based oxides including Mn_3_O_4_ have obvious advantages for a higher battery potential because of a low operating voltage (0.2–0.4 V vs. Li^+^/Li) associated with conversion reactions compared to the iron, cobalt, and nickel-based oxides (0.6–1.2 V)^10^. However, its low electrical conductivity (~ 10^−8^ S/cm to 10^−7^ S/cm) and significant volume change during lithiation/delithiation cause particle collapse and discontinuation of the electrical conduction pathway, leading to serious cycle degradation for initial tens of cycles^11^. Especially in the first cycle, the Coulombic efficiency, defined as the ratio of charge to discharge capacity, tends to be extremely low. This hinders the practical application of Mn_3_O_4_ anodes in future high-energy Li-ion batteries^12^.

Numerous approaches from a microstructure perspective have been proposed to address the intrinsic problems of Mn_3_O_4_ active materials. Surface coating or composites with carbon materials such as graphene^13,14^, graphite^15^, nanotubes/fibers^16–18^, and nanoparticles^19,20^ can improve the low electrical conductivity. Meanwhile, particle morphologies consisting of nanosized^21^, porous^22^, hollow^23^, and hierarchical structures^24,25^ can alleviate volume change. In terms of anode performance, composites of microstructure-controlled Mn_3_O_4_ with carbon materials achieve improved cyclability and a higher capacity. Most of the previous efforts implemented a carbon-supported composite structure^13–18,25^. Mn_3_O_4_ particles on carbon were exposed to electrolytes, making them prone to side reactions involving electrolyte decomposition, which caused the formation of thick solid-electrolyte interphase (SEI) layers during the charge and discharge cycles^26,27^. These SEI layers induced particle detachment from the carbon material or the formation of strongly bonded aggregates, diminishing the synergistic effect of the composite structure. To overcome these challenges, Cai et al. constructed Mn_3_O_4_/C yolk-shell nanorods as a highly improved composite structure^28^, wherein the carbon shell minimizes the formation of the SEI layer and the hollow carbon rods restrict the volume change of Mn_3_O_4_ rods. Thus, the microstructure of Mn_3_O_4_ particles and the composite structure of carbon materials both are important to improve the anode performance.

**Figure 1:**
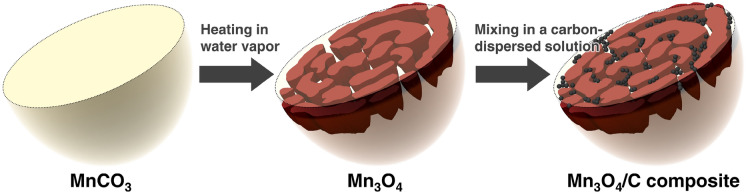
Schematic illustration of the fabrication process of Mn_3_O_4_/C composite.

Herein, we show the carbon nanoparticle-entrapped macroporous Mn_3_O_4_ microsphere as a conversion-type anode morphology for improving cycling stability. The fabrication process is composed of a scalable two-step approach involving the thermal decomposition of MnCO_3_ and mixing in a carbon-dispersed solution, as depicted in Fig. 1. Recently, our group prepared macroporous Mn_3_O_4_ microspheres with unique maze-like pore structures by the thermal decomposition of MnCO_3_ in water vapor^29^. The Mn_3_O_4_ is a porous particle consisting of open macropores. We assessed the applicability of this particle to attain a conversion-type anode material morphology using a single-particle measurement technique^30^. The intrinsic electrochemical performance of the macroporous Mn_3_O_4_ microsphere exhibited a discharge capacity close to the theoretical one, even at an input current equivalent to the 4C-rate, although it decreased gradually due to particle fragmentation. This capacity fading arises from structural changes and insufficient electrical conduction pathways within the Mn_3_O_4_ particle. Therefore, we constructed a carbon network within the macroporous Mn_3_O_4_ microsphere using its open macropores and maze-like pore structure. We inserted and trapped carbon nanoparticles within the particle walls by mixing macroporous Mn_3_O_4_ microspheres in a carbon-dispersed solution. These composites with a simple construction improve cyclability through the following synergistic effects: (1) development of conductive paths within the Mn_3_O_4_ particle^31^; (2) alleviation of volume change by the porous structure; (3) prevention of aggregation by the blocking effect of added carbon nanoparticles^32^. The carbon nanoparticles perform to prevent contact between adjacent Mn_3_O_4_ particle walls due to volume change during charge and discharge cycles. In addition to improving electrical conductivity, the entrapped carbon nanoparticles and their aggregates formed on the composite surface act as buffers during the structural change of Mn_3_O_4_ to Li_2_O and metallic Mn (by the electrochemical conversion reactions) to maintain its unique porous structure. The composite structure and its fabrication process proposed in this study can offer a wide range of applications to other anodes of transition metal oxides due to their simplicity.

## Methodology

### Synthesis of macroporous Mn_3_O_4_ microspheres

Macroporous Mn_3_O_4_ microspheres were synthesized via the thermal decomposition of MnCO_3_ microspheres in water vapor^29^. The spherical MnCO_3_ powder (median size, ~ 3 μm) was prepared using the precipitation method^33^. The prepared MnCO_3_ spheres were thermally decomposed in a tubular furnace equipped with an evaporator. The powder sample was heated to 750 °C at a rate of 5 °C/min and held for 2 h. Deionized water was pumped at a flow rate of 1 mL/min into the evaporator and heated above 100 °C. The generated water vapor was directly introduced into the tubular furnace without any carrier gas and allowed to flow during the holding time. The furnace outlet was opened into the laboratory atmosphere.

### Fabrication of Mn_3_O_4_/C composites

The Mn_3_O_4_/C composites were fabricated from the macroporous Mn_3_O_4_ microspheres and ketjen black carbon (ECP600JD, LION SPECIALTY CHEMICALS Co., Ltd., Japan) nanoparticles as follows. First, carbon powder (3 mg to 30 mg) was added to 2-propanol (30 mL, FUJIFILM Wako Pure Chemical Co., Japan) and dispersed by ultrasonic irradiation for 5 min. Then, the Mn_3_O_4_ powder (90 mg) was added to this carbon-dispersed solution and stirred for 1 h. The treated product was collected by centrifugation, dried at 100 °C, and ground using an agate mortar. Next, the collected powder was added to water and kept overnight to isolate the free carbon. The carbon content in the Mn_3_O_4_/C composites was estimated by weighing the collected free carbon powder.

### Powder characterization

The crystalline phases of the powder samples were characterized by powder X-ray diffraction (XRD, D2 PHASER, Bruker AXS GmbH, Germany) using Cu *K*α radiation generated at 30 kV and 10 mA. The diffraction patterns were acquired in steps of 0.02° (2*θ*) and a counting time of 0.5 s/step. The particle morphologies were examined using scanning electron microscopy (SEM, SU-70, Hitachi Ltd., Japan) and transmission electron microscopy (TEM, JEM-2100F, JEOL Ltd., Japan). The cross-sectional samples were prepared using an Ar ion beam gun (IB-09020CP, JEOL). The particle size distribution of Mn_3_O_4_ was determined using the laser diffraction/scattering method (Microtrac MT3300EXII, NIKKISO Co., Ltd., Japan). Small amounts of the sample were dispersed in a solution of sodium hexametaphosphate (0.05 mass%) using an ultrasonic homogenizer. Its specific surface area (*S*_w_) was estimated using N_2_ adsorption measurements (3Flex, Micromeritics Ltd., USA). Before each measurement, the powder sample was outgassed under vacuum for 3 h at 120 °C. The *S*_w_ of the sample was calculated using the Brunauer–Emmett–Teller (BET) method. Its pore structure was determined using a Hg porosimeter (AutoPore V, micromeritics), while its pore size was calculated using the Washburn equation. The surface tension of Hg was 485 dynes/cm. Contact angles of 130° and 154.9° were used in the cases of bare Mn_3_O_4_ and carbon composites, respectively^34^. Raman spectroscopy (LabRAM ARAMIS, HORIBA Jobin Yvon SAS, France) was performed using the 532 nm excitation line of a He–Ne laser.

### Electrochemical characterization

The anode performance of the fabricated samples was evaluated by using coin-type cells. Bare Mn_3_O_4_ or Mn_3_O_4_/C composites were mixed with acetylene black carbon (Denka Co., Ltd., Japan) and sodium carboxymethyl cellulose (FUJIFILM Wako Pure Chemical) in a weight ratio of 80:12:8 and added a small amount of water. The anode slurry was coated onto a Cu foil using a doctor blade and then dried at 100 °C for 12 h in a vacuum. The dried anode was uniaxially pressed at 40 MPa and punched out of the foil. The active material loadings in the electrodes were about 3.4 mg/cm^2^. A polypropylene membrane (#2400, Celgard LLC, USA) and a 1 M LiPF_6_ (Kishida Chemical Co., Ltd., Japan) solution (30 μL) in a mixture of ethylene carbonate and diethyl carbonate solvents (50:50 vol.%) were used as the separator and the electrolyte, respectively. The 2032-type coin cells were assembled in a glove box filled with dry Ar. The anode performance was characterized at 20 °C using an electrochemical workstation (VMP3, BioLogic Sciences Instruments, France). Cyclic voltammetry (CV) measurements were conducted at a scan rate of 0.2 mV/s within 0–3 V. The charge–discharge tests were performed within 0.01–3 V at varying rates. Electrochemical impedance spectroscopy (EIS) measurements were taken in the frequency range from 10 mHz to 100 kHz with an amplitude of 5 mV.

## Results and discussion

### Materials characterization

**Figure 2:**
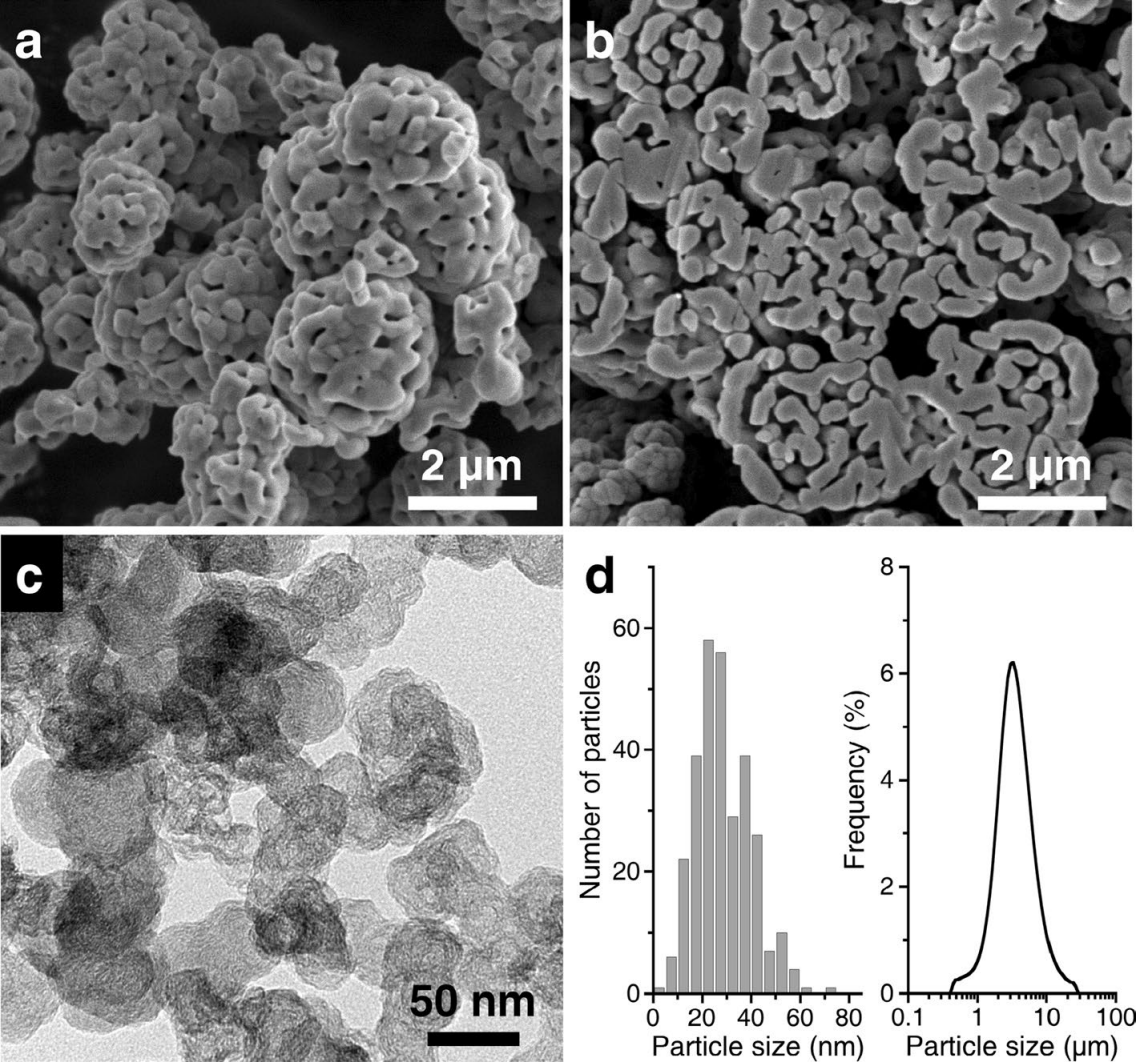
(**a**,**b**) SEM images of macroporous Mn_3_O_4_ microspheres and (**c**) TEM image of carbon nanoparticles. (**d**) Particle size distributions of (left, estimated by TEM) carbon and (right, measured in a liquid) Mn_3_O_4_ powders.

Figure 2 shows the powder characteristics of the prepared Mn_3_O_4_ and carbon nanoparticles used. Open pores enclosed in randomly grown particles are confirmed on the Mn_3_O_4_ microsphere surface (Fig. 2a). A cross-sectional view shows the maze-like pore structure formed by the developed particle walls (Fig. 2b). According to the N_2_ adsorption measurements, the *S*_w_ value of the Mn_3_O_4_ powder is < 3 m^2^/g, indicating the existence of macropores solely. The internal pore size is determined to be ~ 400 nm using Hg porosimetry (more details are provided in the next section). The large open pores allow electrolytes easy access into the particles. When measured in a liquid, the Mn_3_O_4_ powder exhibits a unimodal particle size distribution with almost the same median size as that of MnCO_3_ (Fig. 2d, right). By contrast, the carbon powder possesses a spherical hollow shape and an interconnected chain-like structure (Fig. 2c). The average particle size, estimated from TEM observations, is ~ 35 nm (Fig. 2d, left). The hollow shape provides a high *S*_w_ of 790 m^2^/g. These results reveal that the prepared macroporous Mn_3_O_4_ microspheres have suitable pores for receiving carbon nanoparticles.

### Fabrication and characterization of Mn_3_O_4_/C composites

**Figure 3:**
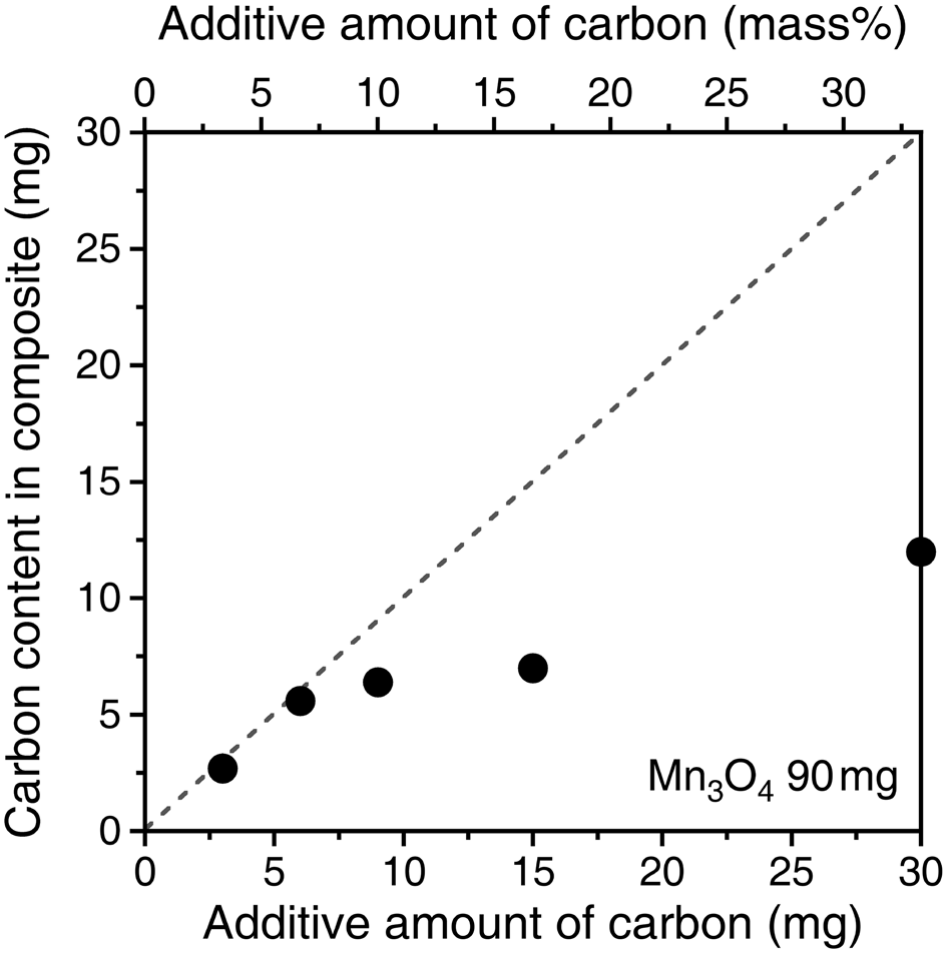
Retained amount of carbon nanoparticles compared to the added amount.

Initially, we investigated the amount of carbon nanoparticles retained within the macroporous Mn_3_O_4_ microspheres. Figure 3 depicts the plot of carbon content in the Mn_3_O_4_/C composites against the added amount. For 90 mg of Mn_3_O_4_, added carbon of up to 6 mg (6.7 mass%) is fully retained in Mn_3_O_4_. A further increase in the amount added exceeds the limit of retention within Mn_3_O_4_. However, upon the addition of 30 mg of carbon (33 mass%), the retention amount indicates an upward trend. In the following experiments, three composite samples (Mn_3_O_4_/C-3, -7, and -33) fabricated with varying amounts of added carbon (3.3 mass%, 6.7 mass%, and 33 mass%) are compared with bare Mn_3_O_4_ particles.

**Figure 4:**
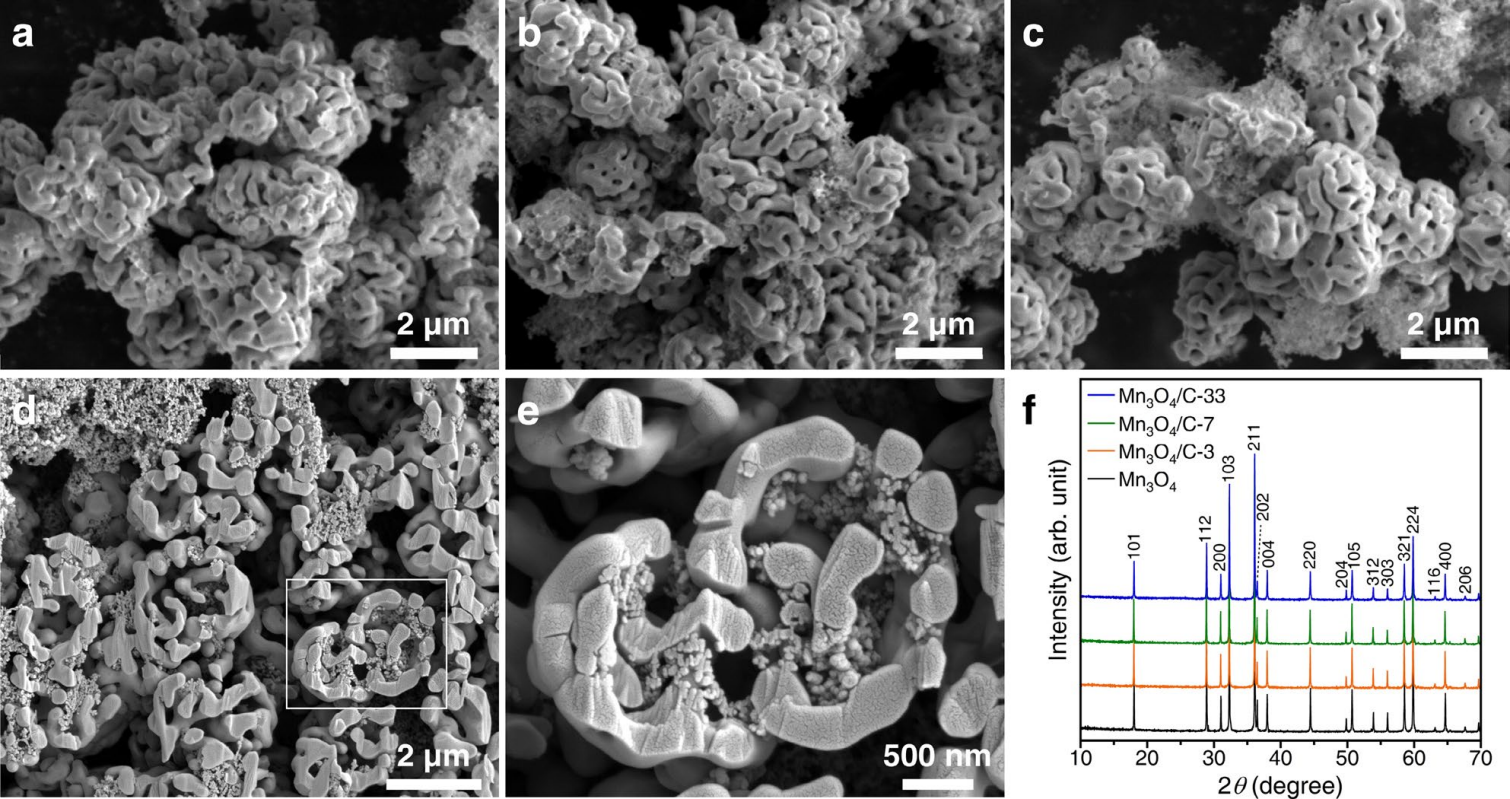
(**a**–**e**) SEM images and (**f**) XRD patterns of the Mn_3_O_4_/C composite samples: (**a**) Mn_3_O_4_/C-3, (**b**,**d**,**e**) Mn_3_O_4_/C-7, and (**c**) Mn_3_O_4_/C-33 samples.

The un-inserted carbon nanoparticles in Mn_3_O_4_ are trapped and retained on the composite surface aggregates. Figure 4 shows the structures and morphologies of the fabricated composites. After insertion in the liquid, the particle morphology of Mn_3_O_4_ remains the same in all the samples (Fig. 4a–c). The carbon nanoparticles are trapped in open macropores on the surface. With an increase in the added amount of carbon, the excess carbon nanoparticles form aggregates on the composite surface due to their chain-like structures (Fig. 4c). This formation of aggregates leads to an increase in the retention amount, as shown in Fig. 3. A cross-sectional view of Mn_3_O_4_/C-7 reveals the capture of carbon nanoparticles within the internal walls of the particle (Fig. 4d,e). The XRD analysis shows that all the composite samples retain the high crystallinity of Mn_3_O_4_ (Fig. 4f). Since the carbon nanoparticles are only entrapped within the Mn_3_O_4_ particles, the crystallinity maintained therein is high compared to that of the yolk-shell^28^ or core-shell^35^ structures fully covered with carbon materials. The composites with carbon nanoparticles exhibited increasing specific surface areas: 17.6, 34.9, and 157 m^2^/g for Mn_3_O_4_/C-3, -7, and -33, respectively.

Furthermore, the structural evaluation was conducted using Raman spectroscopy (Fig. S1). The Raman bands of the prepared Mn_3_O_4_ are consistent with the previous report^36^. Typical carbon peaks located at 1340 cm^−1^ (D band) and 1595 cm^−1^ (G band) are detected in all composite samples. The intensity ratio of the D and G bands (*I*_D_/*I*_G_) for the used ketjen black carbon is approximately 1.1, suggesting a slightly higher proportion of disordered graphitic form (amorphous). Since these two band positions and the *I*_D_/*I*_G_ ratios in all the composite samples are almost the same, no chemical interaction between Mn_3_O_4_ and carbon occurs during the fabrication process.

**Figure 5:**
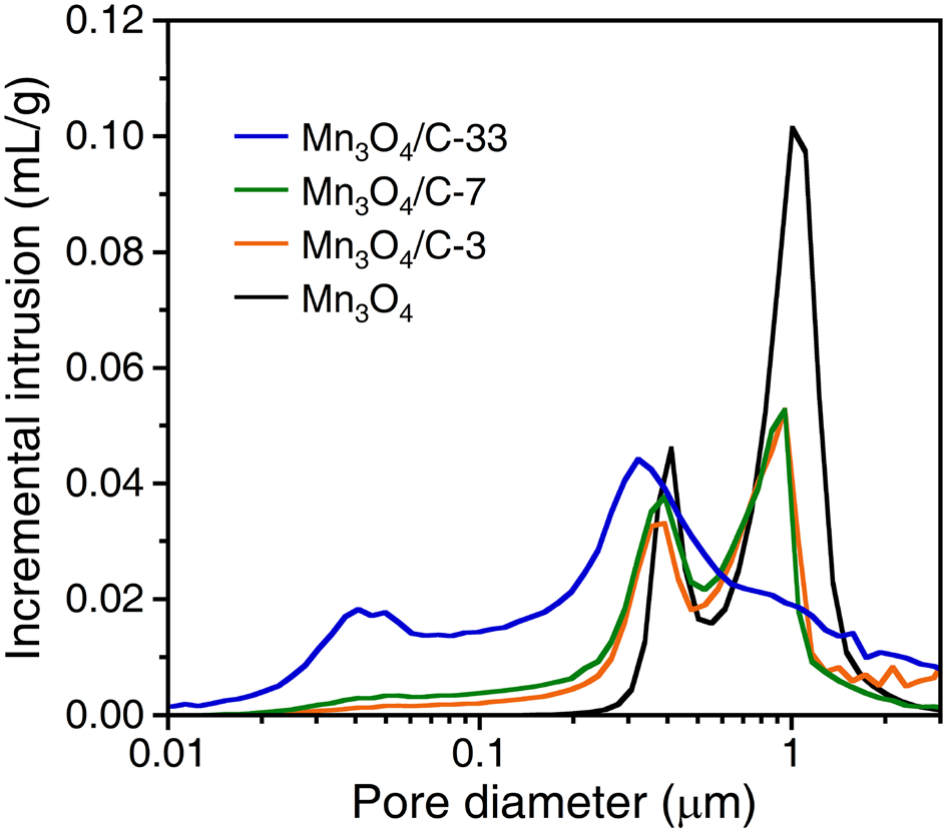
Pore size distribution of the bare Mn_3_O_4_ and Mn_3_O_4_/C composites.

The pore size of Mn_3_O_4_ decreases with the insertion of carbon nanoparticles. Figure 5 shows the pore size distributions of bare Mn_3_O_4_ and Mn_3_O_4_/C composite samples estimated using Hg porosimetry. The pore region at ~ 1 μm is caused by the interparticle spaces between Mn_3_O_4_ microspheres. This pore region disappears for Mn_3_O_4_/C-33 since the formation of carbon aggregates on the surface hinders a close packing. Thus, the pore size of Mn_3_O_4_ is determined to be ~ 400 nm. The size of the macropores keeps diminishing with the insertion of carbon nanoparticles, finalizing as ~ 300 nm for Mn_3_O_4_/C-33. A new pore region appears at 40–50 nm. This nanopore region can be attributed to the carbon aggregates. Since the carbon nanoparticles tend to form interconnected chain-like structures (Fig. 2c), the aggregates spontaneously generate nanopores of several tens of nanometers.

Carbon nanoparticle-entrapped Mn_3_O_4_ microspheres, which differ from the commonly constructed composites, were fabricated via a scalable two-step process. Open macropores enable the direct insertion of conductive nanoparticles by mixing in solution. Templates^37^ or in-situ formations following crystal growth^38^ have been previously implemented to insert conductive materials into the electrode particles. Our process is probably the simplest and most versatile method.

### Electrochemical performance

This study expresses the reduction (lithiation) of Mn_3_O_4_ to Li_2_O and metallic Mn as a charging process and their reverse oxidation (delithiation) to manganese oxides as a discharging process. The cycle performance of the anode is discussed based on its discharge capacities. The C rates (1C = 93.7 mA/g) used in each measurement are calculated from the weight of Mn_3_O_4_ in the composite.

**Figure 6:**
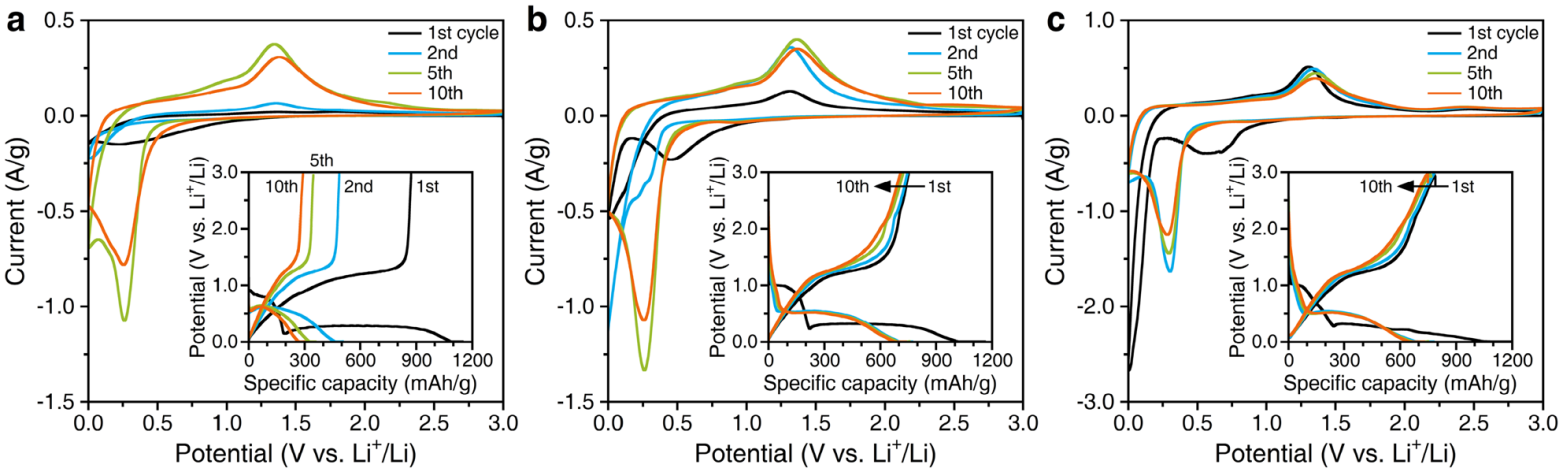
CV curves and charge–discharge curves (inset) of (**a**) bare Mn_3_O_4_, (**b**) Mn_3_O_4_/C-7, and (**c**) Mn_3_O_4_/C-33 composite anodes.

Figure 6 shows the CV and charge–discharge curves of bare Mn_3_O_4_, Mn_3_O_4_/C-7, and Mn_3_O_4_/C-33 composite anodes during the selected cycles (first, second, fifth, and tenth cycles). For the bare Mn_3_O_4_ anode, the CV curve in the first cycle exhibits only a broad reduction peak at ~ 0.25 V (Fig. 6a). This peak disappears in the second CV curve. Instead, a current drop from 0.3 to 0 V occurs. An oxidation peak at 1.35 V can be observed in the anodic scan. The subsequent cycles show the reduction and oxidation peaks at 0.25 V and 1.35 V, respectively. These irreversible initial CV curves indicate unstable electrochemical reactions of bare Mn_3_O_4_^19^. Reflecting this electrochemical instability, the charge and discharge capacities observed at 0.1C decrease drastically with the number of cycles (inset in Fig. 6a). The initial discharge capacity of 873 mAh/g declines to 291 mAh/g after 10 cycles. In contrast, the Mn_3_O_4_/C composite anodes exhibit relatively better reversibility and stability (Fig. 6b,c). The first CV curve for the composites shows a broad reduction peak centered at 0.6–0.5 V and a steep one below 0.2 V in the cathodic scans, and an oxidation peak at 1.3 V in the anodic scans. The principal reduction peak is located at ~ 0.25 V in the subsequent cycles, except for the intensive peak below 0.3 V for Mn_3_O_4_/C-7 in the second cycle. In contrast, the oxidation peak remains at 1.3 V. A small hump at ~ 2.5 V can be observed in the anodic scan for the Mn_3_O_4_/C-33 anode (Fig. S2). In contrast to the electrochemical redox reaction for bare Mn_3_O_4_, the Mn_3_O_4_/C composites exhibit a reversible one, showing improved cyclability during the charge–discharge runs (inset in Fig. 6b,c). The initial discharge capacities of Mn_3_O_4_/C-7 and Mn_3_O_4_/C-33 are 753 mAh/g and 787 mAh/g, respectively. These capacities are maintained even after 10 cycles. The electrochemical performance of the Mn_3_O_4_/C-3 anode lies between that of the bare Mn_3_O_4_ and Mn_3_O_4_/C-7 (Fig. S3). We conclude that the electrochemical performance of the Mn_3_O_4_/C composites improves with increasing carbon content.

The redox reactions during the charge and discharge cycles of Mn_3_O_4_ are discussed based on the above results of the initial electrochemical tests. The initial lithiation of unprocessed Mn_3_O_4_ leads to its reduction to MnO accompanied by the formation of Li_2_O, corresponding to the cathodic peak at 0.6–0.5 V in CV. Besides, the decomposition of electrolytes on the particle surface results in the formation of an SEI layer. The initial charging capacity at a potential plateau of ~ 1.0 V is consumed by these reactions. Another steep cathodic peak at a lower potential is attributed to the further reduction of MnO to metallic Mn, indicating a potential plateau at 0.25 V. The delithiation from Li_2_O and metallic Mn to MnO occurs at 1.3 V. The potential sloping during the discharge process over 2.0 V, especially in the Mn_3_O_4_/C composite, indicates the further oxidation of Mn ions^18,22,23,25^. We have confirmed the crystalline phases of Mn_3_O_4_ and MnO_2_ by XRD from the Mn_3_O_4_/C-33 sample after the electrochemical test (Fig. S4). The formation of MnO_*x*_ (*x* =  ~ 2) with a higher oxidation state than the initial Mn_3_O_4_ has been detected in composites with carbon fibers^18^ or graphene^39^. The formation of MnO_2_ is probably due to the residual oxygen present as defects in the carbon materials^39^. From the second cycle onwards, the reduction reaction to form Li_2_O and metallic Mn shifts to a higher potential; thus, its corresponding plateau is observed at 0.5 V. This is attributed to the following reasons: (i) the formed SEI layer with improved conductivity that enhances the reaction kinetics^19,23^ and (ii) the structural changes during the first lithiation-delithiation process^40^. According to *in operando* X-ray studies, the first lithiation occurs in the sequence Mn_3_O_4_ → LiMn_3_O_4_ → MnO, with the final conversion step involving the reduction of MnO to metallic Mn^41^. The lithiation after the second cycle is reversible without forming the Li-Mn–O intermediate phase. The structural changes in the active material will cause the difference between the first and subsequent lithiation reactions. Herein, some carbon materials in the composites exhibit Li^+^ storage reactions^18^, but the ketjen black carbon used does not show any such behavior. The added carbon nanoparticles contribute to the electrochemical stability of Mn_3_O_4_ without reacting themselves.

**Figure 7:**
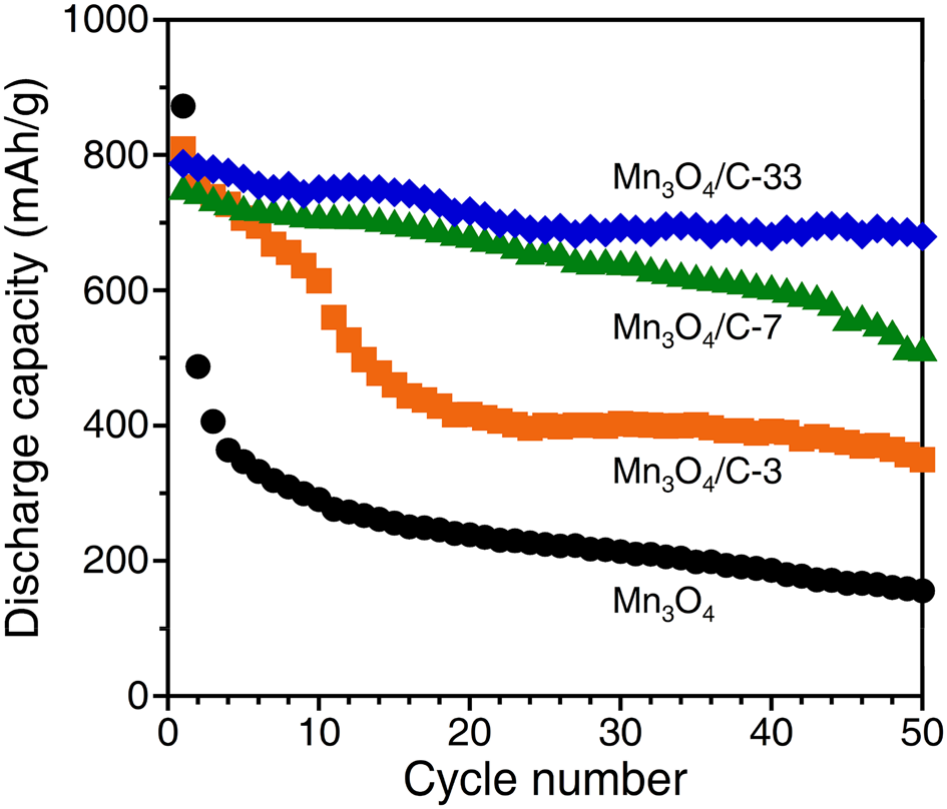
Cycling performance of the bare Mn_3_O_4_ and Mn_3_O_4_/C composite anodes at 0.1C.

We tested the cyclability of the fabricated anodes at 0.1C for up to 50 cycles. Figure 7 plots the discharge capacities recorded for all anodes. As shown in Fig. 6a, the bare Mn_3_O_4_ exhibits a rapid capacity drop during the second cycle. Its discharge capacity retention is only 18% after 50 cycles. For Mn_3_O_4_/C-3, although the discharge capacity stabilizes at ~ 400 mAh/g after the twentieth cycle, its capacity retention eventually drops to 43%. A rapid capacity drop in the initial stage is restrained for the Mn_3_O_4_/C-7 and -33 composite anodes, and consequently, their discharge capacity retention increases to 68% and 86%, respectively. The Mn_3_O_4_/C-33 anode composed of excess carbon exhibits a discharge capacity of 680 mAh/g after 50 cycles. The Coulombic efficiency exhibited more than 60% for the first cycle and remained over 95% during subsequent cycles (Fig. S5). These results suggest a gradual increase in electrochemically inactive regions for bare Mn_3_O_4_ and composites with low carbon contents.

**Figure 8:**
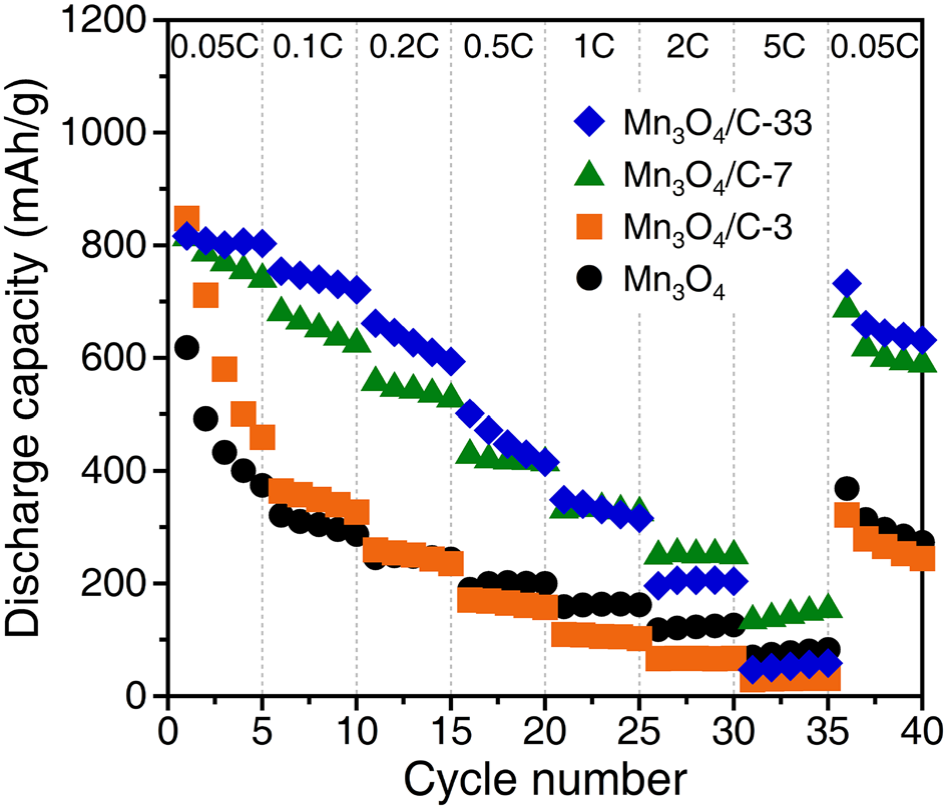
Rate performance of the bare Mn_3_O_4_ and Mn_3_O_4_/C composite anodes.

Furthermore, we investigated the rate performances of all anodes. Figure 8 plots the discharge capacities at different rates ranging from 0.05C to 5C. The bare Mn_3_O_4_ and the Mn_3_O_4_/C-3 anodes exhibit relatively low discharge capacities compared to the composite anodes with high carbon contents at most rates. Meanwhile, the discharge capacities of these two anodes are reversed for the applied current density. A similar tendency is observed with the Mn_3_O_4_/C-7 and -33 anodes; the highest discharge capacity of ~ 150 mAh/g at 5C is obtained with Mn_3_O_4_/C-7. The charge–discharge curves at different rates indicate that the bare Mn_3_O_4_ anode suffers from high internal resistance, shown a voltage drop during the charging process with increasing rates (Fig. S6). The Mn_3_O_4_/C-7 composite anode exhibited a charging plateau at ~ 0.5 V even at higher rates. Increasing the carbon content does not simply improve rate performance. The cycle and rate performances differ depending on the carbon content of the composites. These results suggest that the carbon nanoparticles within the macroporous Mn_3_O_4_ microspheres have both electrochemical and physical merits for improving the anode performance.

### Role of inserted carbon nanoparticles and surface aggregates

**Figure 9:**
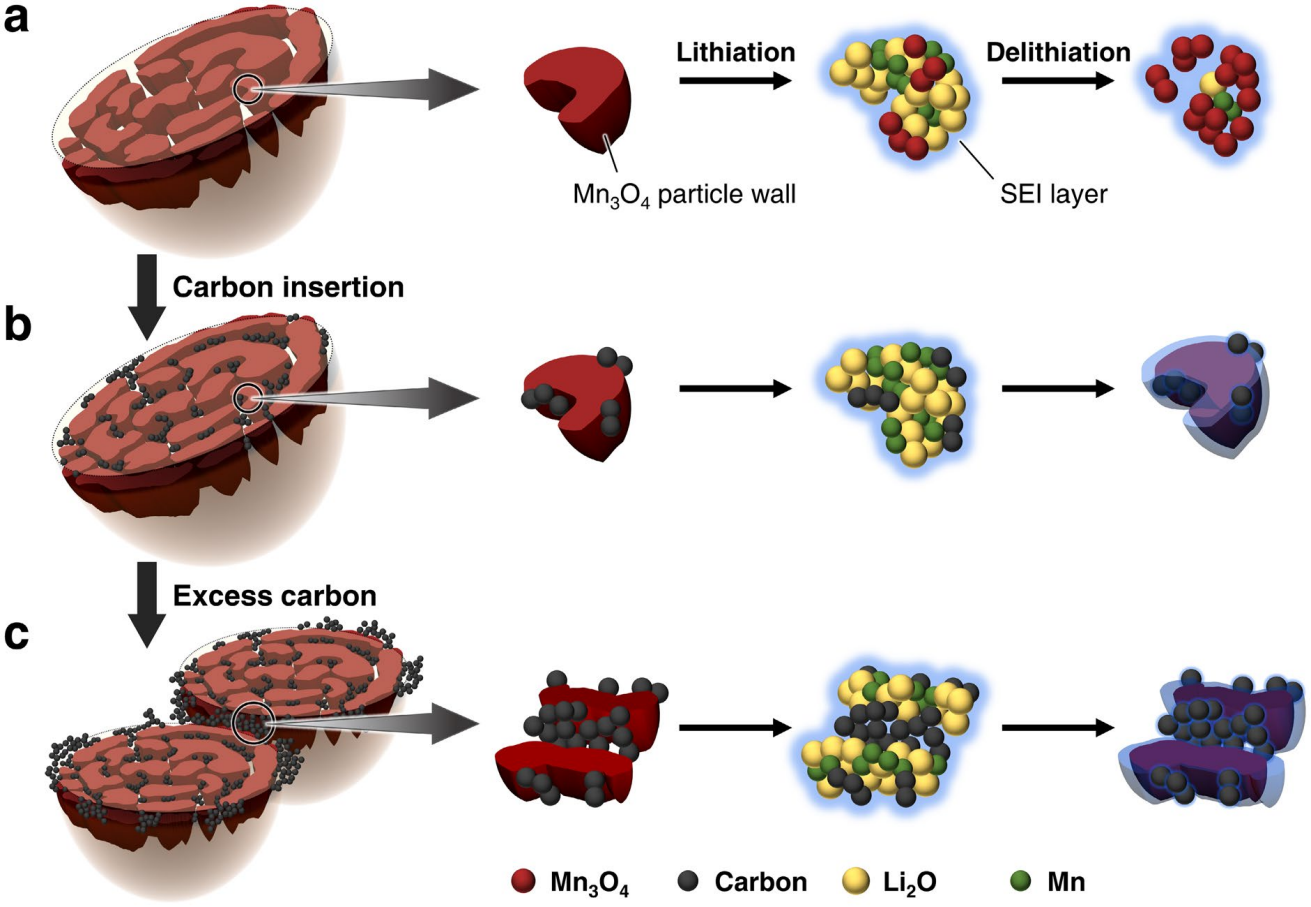
Illustration of the conversion reaction of the (**a**) bare Mn_3_O_4_, (**b**) Mn_3_O_4_/C composite, and (**c**) composite with an excess amount of carbon during the charge–discharge process.

The cyclability of the Mn_3_O_4_ anode improves with the increasing amount of entrapped carbon nanoparticles within the microspheres. The formation of carbon aggregates on the composite surface further enhances its performance. This improved performance of Mn_3_O_4_/C composites can be attributed to the following electrochemical and physical aspects: (1) development of electrical conduction pathways within Mn_3_O_4_ microspheres by the insertion of carbon nanoparticles, (2) retention of the porous structure during the charge–discharge process, and (3) prevention of Mn_3_O_4_ particle aggregation by the surface carbon aggregates. Figure 9 illustrates the effects of carbon nanoparticles on the electrochemical performance and structural stabilization of macroporous Mn_3_O_4_ microspheres during the charge–discharge process.

**Figure 10:**
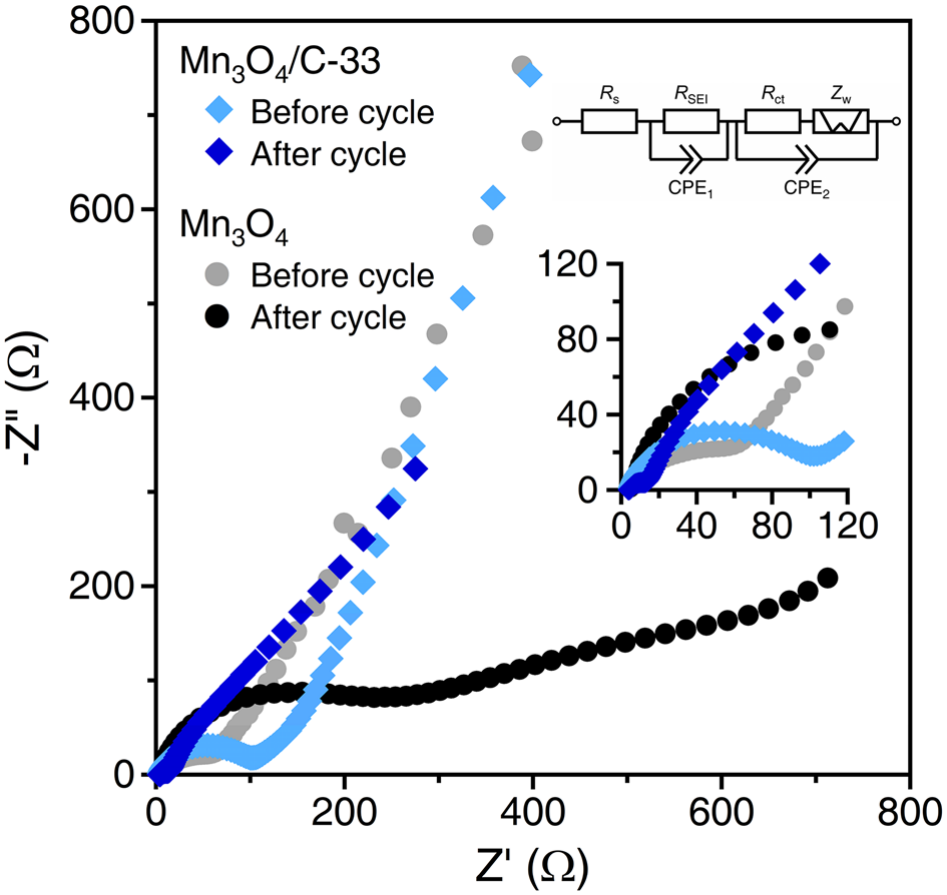
Nyquist plots of bare Mn_3_O_4_ and Mn_3_O_4_/C-33 composite anodes before and after five charge–discharge cycles at 1C.

First, the inserted carbon nanoparticles assume the electrochemical merit as conduction pathways within the Mn_3_O_4_ microspheres. The lithiation process from Mn_3_O_4_ to Li_2_O and metallic Mn, and vice versa, involves particle disproportionation. Electrochemically inactive regions appear in the interior of large particles, such as microspheres, due to interruptions in the conduction pathways (Fig. 9a). This expanse in the irreversible region causes internal resistance, leading to cycle degradation. The conversion reaction can be reversible if a conduction pathway is developed within the microspheres (Fig. 9b). EIS measurements were conducted to clarify the electrochemical merit. Figure 10 shows the EIS spectra of bare Mn_3_O_4_ and Mn_3_O_4_/C-33 composite before and after five charge–discharge cycles. The equivalent circuit is composed of the resistance of the cell system (*R*_s_), including the cell components and electrolyte, the resistance (*R*_SEI_) and capacitance (CPE_1_) of the SEI layer, Faradaic charge-transfer resistance (*R*_ct_), double-layer capacitance (CPE_2_), and the Warburg impedance (*Z*_w_)^42^. Before cycles, the resistances (*R*_ct_) associated with a semicircle in the high-frequency region are 78 Ω and 101 Ω for the bare Mn_3_O_4_ and the Mn_3_O_4_/C-33 composite, respectively. Electrode materials with high surface areas generally exhibit low charge-transfer resistances, but our results show an opposite trend. Such cases are often observed for particles with different surface states^43^, and yolk-shell structures^28,44,45^, in which the active material is fully covered. The present composite, wherein the carbon nanoparticles are entrapped both inside and outside the Mn_3_O_4_ particle, may cause high charge-transfer resistance. After cycles, the bare Mn_3_O_4_ exhibits a high *R*_SEI_ (214 Ω) and *R*_ct_ (631 Ω) in the high- and middle-frequency regions, respectively. In contrast, the Mn_3_O_4_/C-33 shows low *R*_SEI_ (10 Ω) and *R*_ct_ (230 Ω) values. These values indicate activation of the reaction accompanied by improved kinetics. The carbon nanoparticles are incorporated into the SEI layer during the initial charging process, resulting in enhanced electrical conductivity and structural stability of the Mn_3_O_4_ particles^18,19,28^.

Next, the entrapped carbon nanoparticles exert an anchoring effect to retain the porous structure of the Mn_3_O_4_ microspheres. The thick SEI layer formed on the bare Mn_3_O_4_ impedes its return to the original particle morphology. A cross-sectional view of the electrode after ten charge–discharge cycles reveals the collapse of the Mn_3_O_4_ microspheres (Fig. S7). The segregation of the reaction products continues markedly with the expanse of the electrochemically inactive region, as illustrated in Fig. 9a. In contrast, the porous structure in the Mn_3_O_4_/C-33 composite sample is maintained even after 50 cycles (Fig. S7). The carbon nanoparticles within the Mn_3_O_4_ microspheres assist in the reversible conversion reactions on each particle wall. Further, the ketjen black carbon itself does not exhibit Li^+^ storage reactions and thus has the physical merit of restricting the volume change of Mn_3_O_4_ inside the pore space (Fig. 9b). Avoiding conversion reactions between adjacent particle walls prevents product aggregation.

Finally, similar to the entrapped carbon nanoparticles within the Mn_3_O_4_ microspheres, their aggregates on the composite surface also prevent the aggregation of active particles in the electrode (Fig. 9c). According to the Hg porosimetry (measured while applying high pressure), the surface carbon aggregates serve as buffers between the active particles (Fig. 5). The porous structure can be maintained by limiting the volume change during the conversion reaction of the Mn_3_O_4_ anode. The composite structure demonstrated in this study can be applied to other inexpensive transition metal oxide anodes by a further modification with improving electrochemical performance. Liu’s group has designed conversion-type composite anodes with hollow, nanotube, and nanocage structures that exhibited excellent electrochemical performances^46–48^. While allowing for buffering of volume changes, size reduction of porous particles and homogenous carbon coating on the particle wall will attain more improved cycle and rate performances.

## Conclusion

We have constructed a composite structure in which carbon nanoparticles are entrapped within macroporous Mn_3_O_4_ microspheres with maze-like pore structures. As conversion-type anodes for Li-ion batteries, the Mn_3_O_4_/C composites exhibit improved cycle performance with increasing carbon content. The discharge capacity retentions of the bare Mn_3_O_4_ and Mn_3_O_4_/C-33 anodes after 50 cycles are 18% and 86%, respectively. Carbon nanoparticles are entrapped internally through the open macropores of the Mn_3_O_4_ microspheres and form aggregates on the composite surface when added in excess amounts. This morphology of the carbon nanoparticles assists in improving the cycle performance of Mn_3_O_4_ both electrochemically and physically. The present composite structure can be fabricated via a scalable two-step process that involves preparing macroporous microspheres of active materials and mixing them in a carbon-dispersed solution. The demonstrated composite structure and its fabrication process can be applied to other conversion-type anode materials that suffer from cycle degradation, including inexpensive transition metal oxides (Mn_2_O_3_, Fe_2_O_3_, and Fe_3_O_4_) and sulfides. Moreover, this study will enable the incorporation of future applied materials in applications such as energy storage, environmental filtration, and selective chemical reaction by utilizing the unique pore structure of the Mn_3_O_4_ microspheres.

## Supplementary Information

Supplementary Figures.

## Data Availability

All data included in this study are available upon request by contact with the corresponding author.

## References

[1] Zhang L, Zhou Q, Liu Z, Hou X, Li Y, Lv Y (2009). Novel Mn_3_O_4_ Micro-octahedra: Promising cataluminescence sensing material for acetone. Chem. Mater..

[2] Bigiani L, Maccato C, Carraro G, Gasparotto A, Sada C, Comini E, Barreca D (2018). Tailoring vapor-phase fabrication of Mn_3_O_4_ nanosystems: From synthesis to gas-sensing applications. ACS Appl. Nano Mater..

[3] Fei, Z.-Y., Sun, B., Zhao, L., Ji, W.-J. & Au, C.-T. Strong morphological effect of Mn_3_O_4_ nanocrystallites on the catalytic activity of Mn_3_O_4_ and Au/Mn_3_O_4_ in benzene combustion. *Chem*. −*Eur*. *J*. **19**, 6480–6487 (2013). 10.1002/chem.201204112 23526641

[4] Pan H, Jian Y, Chen C, He C, Hao Z, Shen Z, Liu H (2017). Sphere-shaped Mn_3_O_4_ catalyst with remarkable low-temperature activity for methyl−ethyl−ketone combustion. Environ. Sci. Technol..

[5] Dong R, Ye Q, Kuang L, Lu X, Zhang Y, Zhang X, Tan G, Wen Y, Wang F (2013). Enhanced supercapacitor performance of Mn_3_O_4_ nanocrystals by doping transition-metal ions. ACS Appl. Mater. Interfaces.

[6] Seo WS, Jo HH, Lee K, Kim B, Oh SJ, Park JT (2004). Size-dependent magnetic properties of colloidal Mn_3_O_4_ and MnO nanoparticles. Angew. Chem. Int. Ed..

[7] Yu T, Moon J, Park J, Park YI, Na HB, Kim BH, Song IC, Moon WK, Hyeon T (2009). Various-shaped uniform Mn_3_O_4_ nanocrystals synthesized at low temperature in air atmosphere. Chem. Mater..

[8] Poizot P, Laruelle S, Grugeon S, Dupont L, Tarascon J-M (2000). Nano-sized transition-metal oxides as negative-electrode materials for lithium-ion batteries. Nature.

[9] Pasero D, Reeves N, West AR (2005). Co-doped Mn_3_O_4_: A possible anode material for lithium batteries. J. Power Sources.

[10] Deng Y, Wan L, Xie Y, Qin X, Chen G (2014). Recent advances in Mn-based oxides as anode materials for lithium ion batteries. RSC Adv..

[11] Lee SH, Yu S-H, Lee JE, Jin A, Lee DJ, Lee N, Jo H, Shin K, Ahn T-Y, Kim Y-W, Choe H, Sung Y-E, Hyeon T (2013). Self-assembled Fe_3_O_4_ nanoparticle clusters as high-performance anodes for lithium ion batteries via geometric confinement. Nano Lett..

[12] Yu S-H, Lee SH, Lee DJ, Sung Y-E, Hyeon T (2016). Conversion reaction-based oxide nanomaterials for lithium ion battery anodes. Small.

[13] Wang H, Cui L-F, Yang Y, Casalongue HS, Robinson JT, Liang Y, Cui Y, Dai H (2010). Mn_3_O_4_−graphene hybrid as a high-capacity anode material for lithium ion batteries. J. Am. Chem. Soc..

[14] Nam I, Kim ND, Kim G-P, Park J, Yi J (2013). One step preparation of Mn_3_O_4_/graphene composites for use as an anode in Li ion batteries. J. Power Sources.

[15] Liu S-Y, Xie J, Zhen Y-X, Cao G-S, Zhu T-J, Zhao X-B (2012). Nanocrystal manganese oxide (Mn_3_O_4_, MnO) anchored on graphite nanosheet with improved electrochemical Li-storage properties. Electrochim. Acta.

[16] Cui X, Hu F, Wei W, Chen W (2011). Dense and long carbon nanotube arrays decorated with Mn_3_O_4_ nanoparticles for electrodes of electrochemical supercapacitors. Carbon.

[17] Luo, S., Wu, H., Wu, Y., Jiang, K., Wang. J. & Fan, S. Mn_3_O_4_ Nanoparticles anchored on continuous carbon nanotube network as superior anodes for lithium ion batteries. *J*. *Power Sources* **249**, 463–469 (2014).

[18] Ma F, Yuan A, Xu J (2014). Nanoparticulate Mn_3_O_4_/VGCF composite conversion-anode material with extraordinarily high capacity and excellent rate capability for lithium ion batteries. ACS Appl. Mater. Interfaces.

[19] Wang C, Yin L, Xiang D, Qi Y (2012). Uniform carbon layer coated Mn_3_O_4_ nanorod anodes with improved reversible capacity and cyclic stability for lithium ion batteries. ACS Appl. Mater. Interfaces.

[20] Nagamuthu S, Vijayakumar S, Muralidharan G (2013). Synthesis of Mn_3_O_4_/amorphous carbon nanoparticles as electrode material for high performance supercapacitor applications. Energy Fuels.

[21] Gao J, Lowe MA, Abruña HD (2011). Spongelike nanosized Mn_3_O_4_ as a high-capacity anode material for rechargeable lithium batteries. Chem. Mater..

[22] Bai Z, Zhang X, Zhang Y, Guo C, Tang B (2014). Facile synthesis of mesoporous Mn_3_O_4_ nanorods as a promising anode material for high performance lithium-ion batteries. J. Mater. Chem. A.

[23] Jian G, Xu Y, Lai L-C, Wang C, Zachariah MR (2014). Mn_3_O_4_ hollow spheres for lithium-ion batteries with high rate and capacity. J. Mater. Chem. A.

[24] Su H, Xu Y-F, Feng S-C, Wu Z-G, Sun X-P, Shen C-H, Wang J-Q, Li J-T, Huang L, Sun S-G (2015). Hierarchical Mn_2_O_3_ hollow microspheres as anode material of lithium ion battery and its conversion reaction mechanism investigated by XANES. ACS Appl. Mater. Interfaces.

[25] Tang C, Xiong F, Yao X, Tan S, Lan B, An Q, Luo P, Mai L (2019). Hierarchical Mn_3_O_4_/graphene microflowers fabricated via a selective dissolution strategy for alkali-metal-ion storage. ACS Appl. Mater. Interfaces.

[26] Liu N, Lu Z, Zhao J, McDowell MT, Lee H-W, Zhao W, Cui Y (2014). A pomegranate-inspired nanoscale design for large-volume-change lithium battery anodes. Nat. Nanotech..

[27] Mao W, Yue W, Xu Z, Wang J, Zhang J, Li D, Zhang B, Yang S, Dai K, Liu G, Ai G (2020). Novel hoberman sphere design for interlaced Mn_3_O_4_@CNT architecture with atomic layer deposition-coated TiO_2_ overlayer as advanced anodes in li-ion battery. ACS Appl. Mater. Interfaces.

[28] Cai Z, Xu L, Yan M, Han C, He L, Hercule KM, Niu C, Yuan Z, Xu W, Qu L, Zhao K, Mai L (2015). Manganese oxide/carbon yolk−shell nanorod anodes for high capacity lithium batteries. Nano Lett..

[29] Kozawa T (2018). Preparation of macroporous Mn_3_O_4_ microspheres via thermal decomposition in water vapor. ChemistrySelect.

[30] Kozawa T, Nishikawa K (2020). Macroporous Mn_3_O_4_ microspheres as a conversion-type anode material morphology for li-ion batteries. J. Solid State Electrochem..

[31] Zhang X, Ju Z, Zhu Y, Takeuchi KJ, Takeuchi ES, Marschilok AC, Yu G (2021). Multiscale understanding and architecture design of high energy/power lithium-ion battery electrodes. Adv. Energy Mater..

[32] Jiang Y, Yue J-L, Guo Q, Xia Q, Zhou C, Feng T, Xu J, Xia H (2018). Highly porous Mn_3_O_4_ micro/nanocuboids with in situ coated carbon as advanced anode material for lithium-ion batteries. Small.

[33] Kozawa T, Yanagisawa K, Murakami T, Naito M (2016). Growth behavior of LiMn_2_O_4_ particles formed by solid-state reactions in air and water vapor. J. Solid State Chem..

[34] León y León, C.A. New perspectives in mercury porosimetry. *Adv*. *Colloid Interface Sci*. **76–77**, 341–372 (1998).

[35] Sun B, Chen Z, Kim H-S, Ahn H, Wang G (2011). MnO/C core-shell nanorods as high capacity anode materials for lithium-ion batteries. J. Power Sources.

[36] Julien CM, Massot M, Poinsignon C (2004). Lattice vibrations of manganese oxides: Part I periodic structures. Spectrochim. Acta Part A.

[37] Reddy ALM, Shaijumon MM, Gowda SR, Ajayan PM (2009). Coaxial MnO_2_/carbon nanotube array electrodes for high-performance lithium batteries. Nano Lett..

[38] Huang G, Zhang F, Du X, Qin Y, Yin D, Wang L (2015). Metal organic frameworks route to in situ insertion of multiwalled carbon nanotubes in Co_3_O_4_ polyhedra as anode materials for lithium-ion batteries. ACS Nano.

[39] Kim H, Kim S-W, Hong J, Park Y-U, Kang K (2011). Electrochemical and Ex-situ analysis on manganese oxide/graphene hybrid anode for lithium rechargeable batteries. J. Mater. Res..

[40] Guo J, Liu Q, Wang C, Zachariah MR (2012). Interdispersed amorphous MnO_*x*_–carbon nanocomposites with superior electrochemical performances as lithium-storage material. Adv. Funct. Mater..

[41] Lowe MA, Gao J, Abruña HD (2013). In operando X-ray studies of the conversion reaction in Mn_3_O_4_ lithium battery anodes. J. Mater. Chem. A.

[42] Andre, D., Meiler, M., Steiner, K., Walz, H., Soczka-Guth, T. & Sauer, D. U. Characterization of high-power lithium-ion batteries by electrochemical impedance spectroscopy. II: Modelling. *J*. *Power Sources* **196**, 5349–5356 (2011).

[43] Shaju, K. M. & Bruce, P. G. Nano-LiNi_0.5_Mn_1.5_O_4_ Spinel: A high power electrode for Li-ion batteries. *Dalton Trans*. 5471–5475 (2008). 10.1039/b806662k 19082030

[44] Choi SH, Kang YC (2013). Yolk-shell, hollow, and single-crystalline ZnCo_2_O_4_ powders: Preparation using a simple one-pot process and application in lithium-ion batteries. Chemsuschem.

[45] Won JM, Choi SH, Hong YJ, Ko YN, Kang YC (2014). Electrochemical properties of yolk-shell structured ZnFe_2_O_4_ powders prepared by a simple spray drying process as anode material for Lithium-ion battery. Sci. Rep..

[46] Liu, J., Wan, Y., Liu, C., Liu, W., Ji, S., Zhou, Y. & Wang, J. Solvothermal synthesis of uniform Co_3_O_4_/C hollow quasi-nanospheres for enhanced lithium ion intercalation applications. *Eur*. *J*. *Inorg*. *Chem*. 3825–3829 (2012).

[47] Liu J, Xu X, Hu R, Yang L, Zhu M (2016). Uniform hierarchical Fe_3_O_4_@Polypyrrole nanocages for superior lithium ion battery anodes. Adv. Energy Mater..

[48] Xu X, Shen J, Li F, Wang Z, Zhang D, Zuo S, Liu J (2020). Fe_3_O_4_@C nanotubes grown on carbon fabric as a free-standing anode for high-performance Li-ion batteries. Chem. Eur. J..

