# Supplementary material for: Carbon nanoparticle-entrapped macroporous Mn3O4 microsphere anodes with improved cycling stability for Li-ion batteries
Source: Sci Rep. 2022 Jul 14;12:11992. doi: 10.1038/s41598-022-16383-0 (PMC9283411; doi:10.1038/s41598-022-16383-0)
Supplement: Supplementary file 1 — Supplementary Figures. [file 41598_2022_16383_MOESM1_ESM.pdf]

Supplementary Information

Carbon Nanoparticle-Entrapped Macroporous Mn_3O_4 Microsphere Anodes with Improved Cycling Stability for Li-Ion Batteries

Takahiro Kozawa, Fumiya Kitabayashi, Kayo Fukuyama, Makio Naito*

Joining and Welding Research Institute, Osaka University, 11-1 Mihogaoka, Ibaraki, Osaka 567-0047, Japan

*Corresponding author E-mail: t-kozawa@jwri.osaka-u.ac.jp

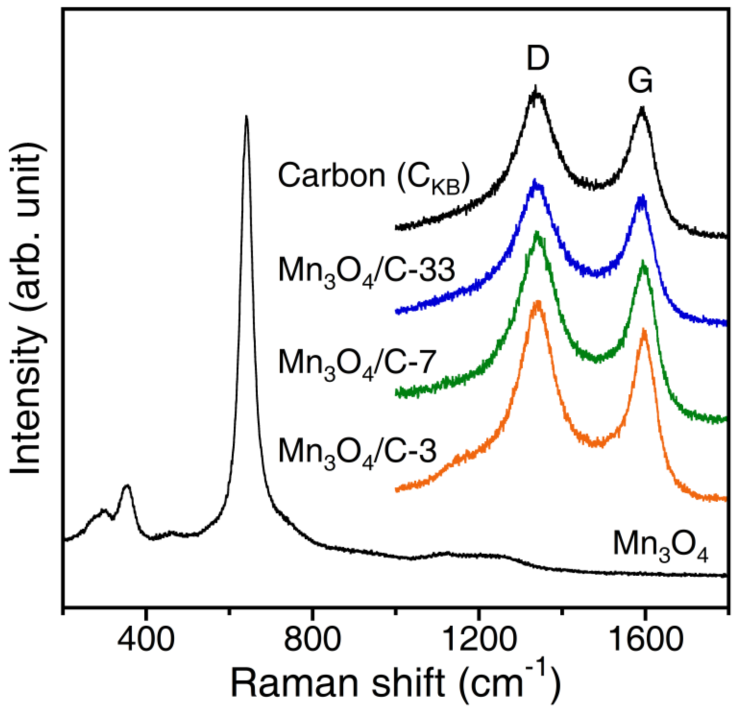

Figure S1. Raman spectra of bare Mn_3O_4, Mn_3O_4/C composites, and ketjen black carbon (C_{KB}) samples.

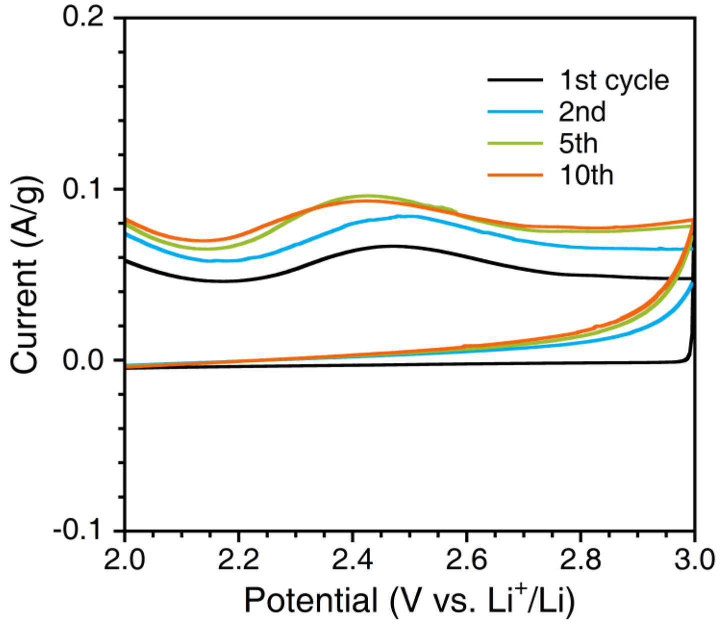

Figure S2. CV curves of the Mn_3O_4/C-33 composite anode.

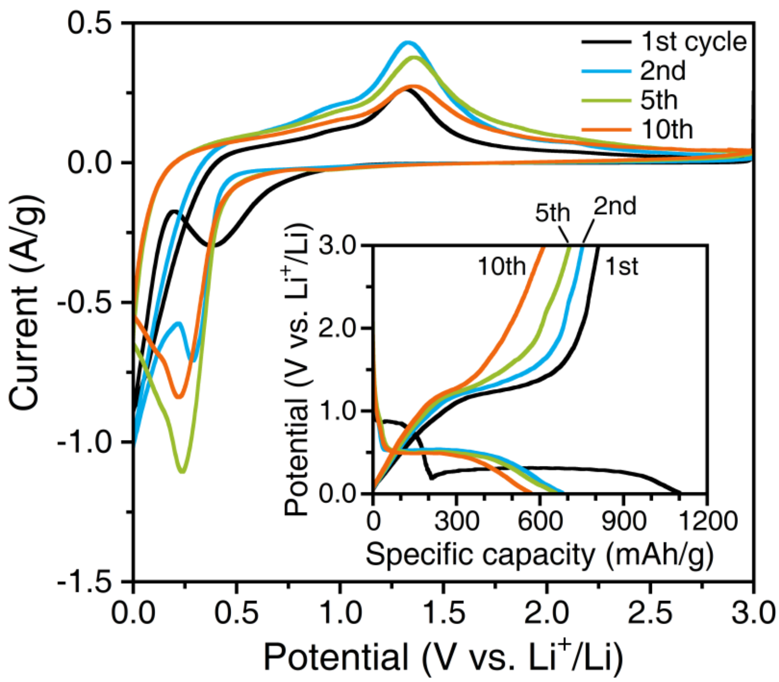

Figure S3. CV curves and charge-discharge curves (inset) of the Mn_3O_4/C-3 composite anode.

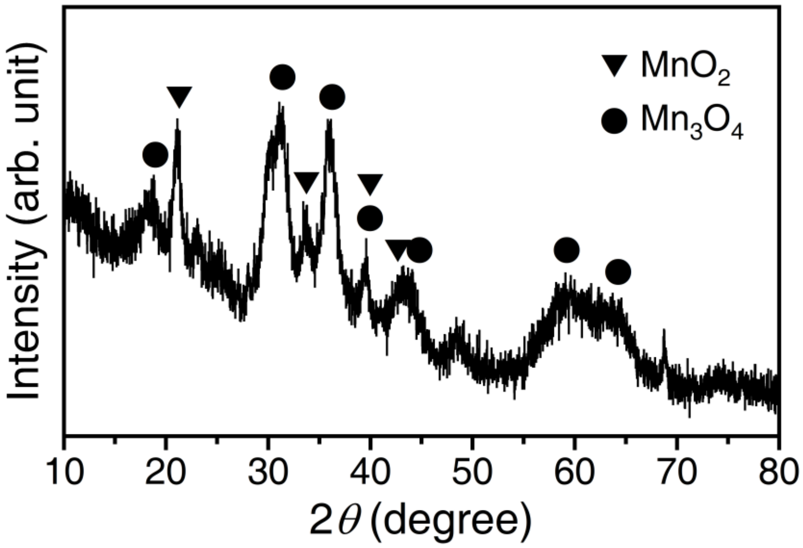

Figure S4. XRD pattern of the Mn_3O_4/C-33 anode after the electrochemical test.

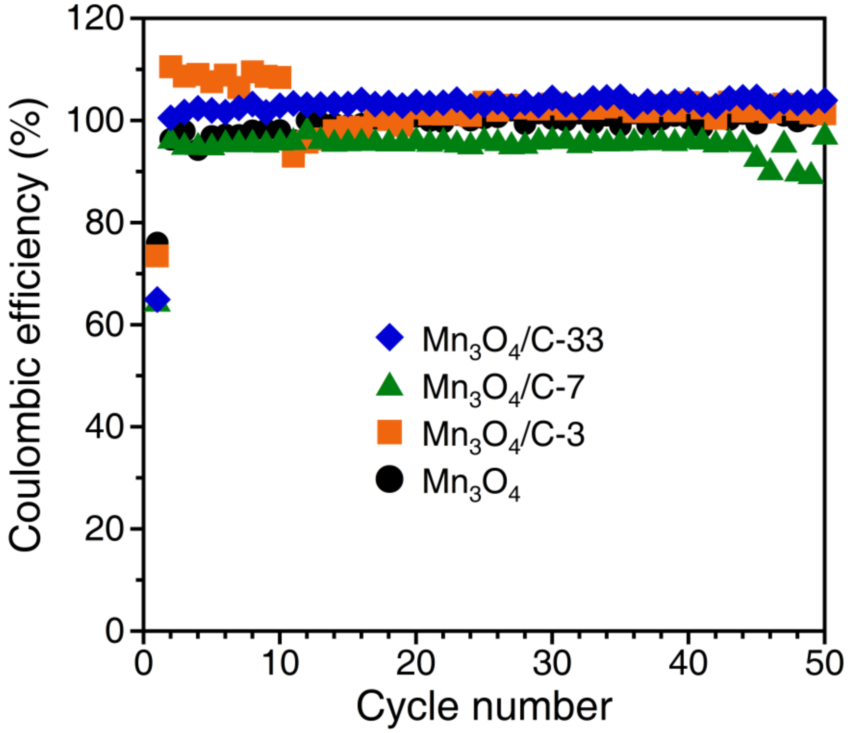

Figure S5. Coulombic efficiency for the cycling tests of the bare Mn_3O_4 and Mn_3O_4/C composite anodes.

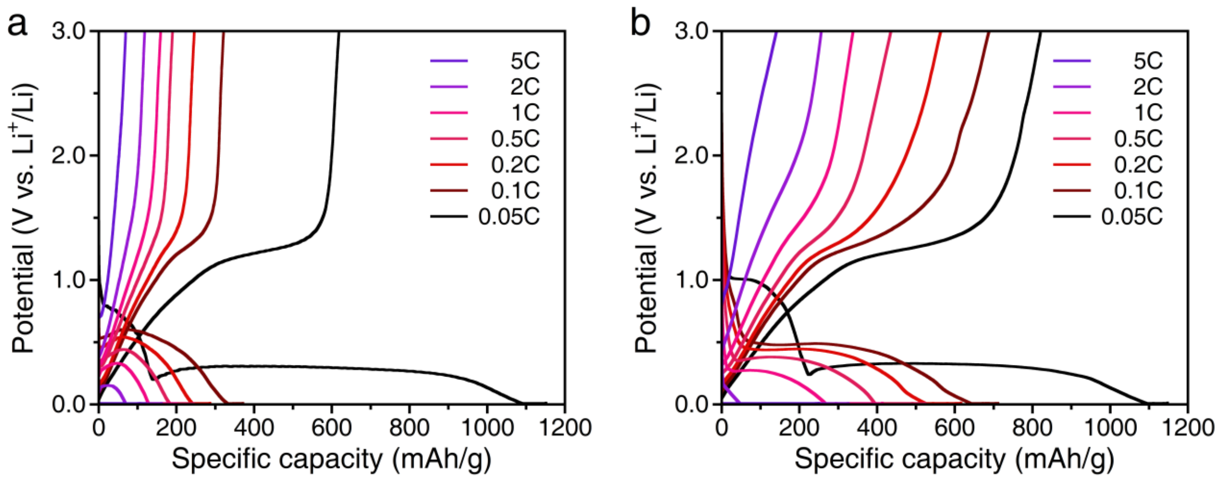

Figure S6. Charge-discharge curves of (a) bare Mn_3O_4 and (b) Mn_3O_4/C-7 composite anodes at different rates.

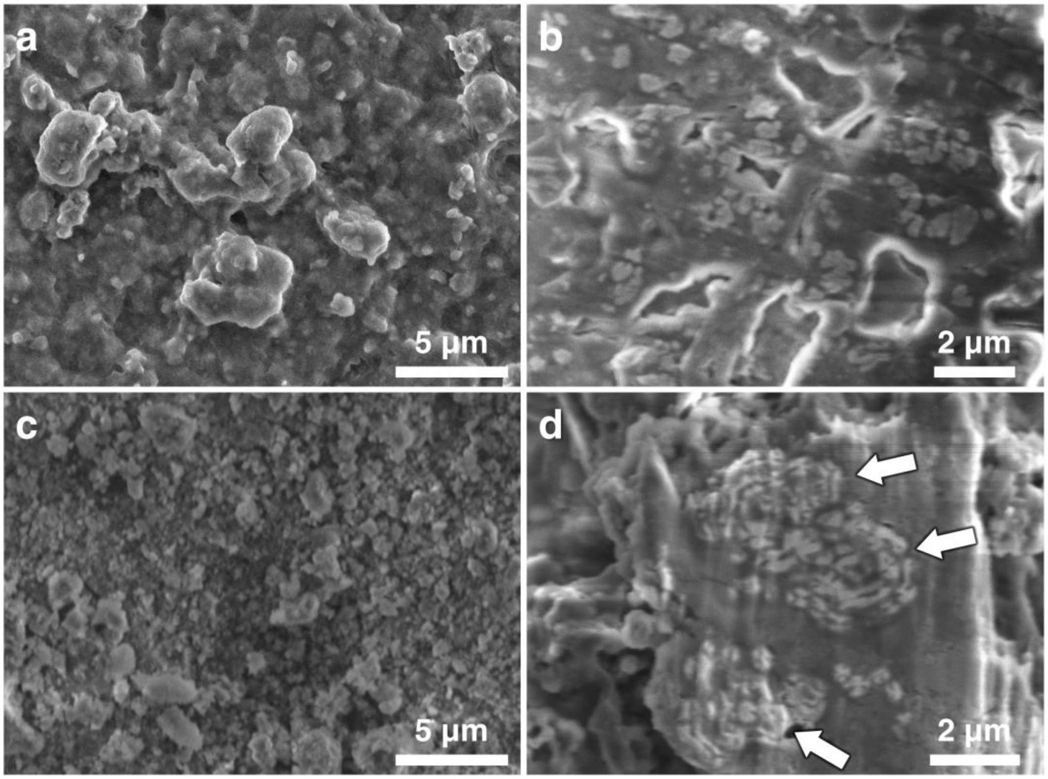

Figure S7. SEM images of (a, b) bare Mn_3O_4 and (c, d) Mn_3O_4/C-33 anodes after the electrochemical test: (b, d) cross-sectional views.
